# U. S. V. M. A.

**Published:** 1895-05

**Authors:** 


### U. S. V. M. A.

*To the Members of the United States Veterinary Medical Association and the Profession throughout the United States.*

The Comitia Minora of the above Association have decided to hold the annual meeting of 1895, in September next, at Des Moines, Iowa. It is earnestly requested that all those desiring a place upon the programme will early communicate with the Secretary. All State and local associations will at their coming meetings elect delegates without further notice. By order of the President.

W. HORACE HOSKINS,
President.
LEONARD PEARSON,
Secretary.
2024 Pine St., Philadelphia.

Tuberculosis will again be a prominent topic at the coming meeting of the U. S. V. M. A. The position of the Western people on this subject will be considered by Western veterinarians.

Eleven votes were cast for Des Moines, 1 vote for Columbus, Ohio, and 2 for Buffalo, if the meeting was to be held that far east, and for Des Moines if Buffalo was out of the race. The plea for Des Moines was the strongest ever put forth to secure the meeting, and bodes well for the success of the decision.

How many Eastern associations will send Delegates to Des Moines?

How many Eastern veterinarians will plan to attend?

The success of this meeting will largely determine how far west future meetings may well be taken and whether we will go to California in 1897.

Treasurer Robertson sounded the keynote for California two years ago for 1897. How many will follow in the movement?

The past year's experience in dealing with the many important aspects of the tuberculosis question from a practical standpoint should bear rich fruits at the coming meeting of the U. S. V. M. A.

Every State that has taken up the subject of bovine tuberculosis should send her strongest representation to Des Moines, where every phase of the question might be thoroughly considered and thus bring forth some uniform plan toward which all future legislation might tend.

Should the one-and-one-third fare be granted by the railroads to Des Moines the fare from Philadelphia quoted by the Baltimore and Ohio will be $36.20

We are promised at Des Moines the attendance of several army veterinarians. Also a delegate from the California State Veterinary Medical Association.

Buffalo will be an easy winner for the meeting in 1896. She deserves it. She has some of the most progressive and earnest association workers in the country.

Iowa has long had one of the most earnest and successful State Associations in our country. They did yeoman service in using their influence to secure the '95 meeting for their State.

Every adjoining State Association, the Missouri Valley organization, and even California fell into line in the enthusiastic desire that the U. S. V. M. A. should this year go as far west at least as Des Moines, Iowa.

Pennsylvania will be represented at Des Moines by delegates from her State and local Associations.

Vice-President Turner believes that it will be the strongest move yet to complete the thorough amalgamation of the profession East and West.

The new date in September will bring representation from almost every experimental station.

